# The prediction-error hypothesis of schizophrenia: new data point to circuit-specific changes in dopamine activity

**DOI:** 10.1038/s41386-021-01188-y

**Published:** 2021-09-29

**Authors:** Samuel J. Millard, Carrie E. Bearden, Katherine H. Karlsgodt, Melissa J. Sharpe

**Affiliations:** 1 Department of Psychology, University of California, Los Angeles, CA 90095 USA; 2 Department of Psychiatry and Biobehavioral Sciences, University of California, Los Angeles, CA 90095 USA

**Keywords:** Cognitive neuroscience, Schizophrenia

## Abstract

Schizophrenia is a severe psychiatric disorder affecting 21 million people worldwide. People with schizophrenia suffer from symptoms including psychosis and delusions, apathy, anhedonia, and cognitive deficits. Strikingly, schizophrenia is characterised by a learning paradox involving difficulties learning from rewarding events, whilst simultaneously ‘overlearning’ about irrelevant or neutral information. While dysfunction in dopaminergic signalling has long been linked to the pathophysiology of schizophrenia, a cohesive framework that accounts for this learning paradox remains elusive. Recently, there has been an explosion of new research investigating how dopamine contributes to reinforcement learning, which illustrates that midbrain dopamine contributes in complex ways to reinforcement learning, not previously envisioned. This new data brings new possibilities for how dopamine signalling contributes to the symptomatology of schizophrenia. Building on recent work, we present a new neural framework for how we might envision specific dopamine circuits contributing to this learning paradox in schizophrenia in the context of models of reinforcement learning. Further, we discuss avenues of preclinical research with the use of cutting-edge neuroscience techniques where aspects of this model may be tested. Ultimately, it is hoped that this review will spur to action more research utilising specific reinforcement learning paradigms in preclinical models of schizophrenia, to reconcile seemingly disparate symptomatology and develop more efficient therapeutics.

## Introduction

### Moving towards a cohesive understanding of how differences in dopamine signalling in discrete circuits could contribute to the paradoxical symptoms of schizophrenia

Schizophrenia is a severe debilitating psychiatric disorder that affects 21 million people, with a prevalence of around 1% worldwide [1, 2]. People with schizophrenia experience an unemployment rate of ~80–90% [3, 4], and a 15–20 year shorter life expectancy compared to the general population [2, 5–7]. The disorder is characterised by a set of core features, including hallucinations and delusions (i.e., positive symptoms), apathy, anhedonia, avolition (i.e., negative symptoms), and cognitive deficits [8–10]. Reinforcement learning deficits in particular are strongly linked to the development of both positive and negative symptoms, and are often present in first episode and frank psychosis, and in populations at risk of developing psychosis [11–15]. Aberrant dopaminergic signalling has long been linked to the pathophysiology of schizophrenia [16–21]. Indeed, the primary mechanism of action of current atypical antipsychotics is contingent upon reducing activity at the D2 receptor [22]. Whilst these antipsychotics are somewhat effective in the treatment of positive symptoms of schizophrenia, they are accompanied by intolerable side effects [23], and cognitive impairments are often unaffected or worsened [24]. Therefore, there is an urgent need to develop our understanding of cognitive dysfunction in schizophrenia to guide alternative therapeutics [24].

The fact that current antipsychotic treatments targeting dopamine activity alleviate positive symptoms, but do not generally impact significantly on negative symptoms or cognitive impairments, illustrates the difficulties that we face in trying to develop a cohesive understanding of the neural basis of schizophrenia. The complexity of this disorder is also reflected in the learning paradox that we see in people with schizophrenia. Specifically, patients show an increase in learning about irrelevant information (correlated with positive symptoms), and a decrease in learning about reward-predictive information (correlated with negative/cognitive symptoms; [25–30]). While there has been recent interest in thinking about how nuanced changes in subcortical dopamine might contribute to schizophrenia symptomatology [27, 31], it is generally difficult to explain this dissociation within existing models of reinforcement learning. As a result, the field still lacks a coherent framework that can help account for this learning paradox seen in schizophrenia.

Recent research emerging from basic neuroscience may be able to help us to refine models of how changes in dopamine circuits could produce the learning paradox seen in schizophrenia. Specifically, this research has demonstrated that we can no longer explain phasic dopamine signalling as a homogenous signal that broadcasts salience or value of a current event [32–43]. It is now believed that dopamine signalling can function in qualitatively different ways in different neural circuits to produce learning in many different situations [35, 44], not previously envisioned by traditional theories of dopamine function [45–48]. From this emerges the exciting possibility that a change in the balance of inputs and outputs to the dopamine system could produce the paradoxical changes in learning seen in schizophrenia, without the need to appeal to other neurotransmitter systems or dissociative effects in subcortical and prefrontal areas. Accordingly, in this review, we make the case for how we might envision specific changes in particular dopamine circuits as contributing to the reinforcement-learning paradox seen in schizophrenia, building on recent works that have begun to conceptualise a more nuanced role for dopamine in schizophrenia [27, 49]. As such, this is a “call to action” to utilise cutting-edge basic neuroscience techniques in the context of reinforcement learning to investigate circuit-defined neural changes in preclinical models of schizophrenia. It is our hope that this will provide a new direction for developing therapeutics that target particular dopaminergic circuits to simultaneously alleviate positive and negative symptoms and cognitive deficits associated with schizophrenia.

## The reinforcement-learning deficits and their neural correlates

The first studies that revealed deficits in reinforcement learning in people with schizophrenia demonstrated enhancements in learning about irrelevant stimuli, or neutral information, which healthy controls usually ignore. This is manifest by failures of latent inhibition, overshadowing, blocking, and learned irrelevance tasks. Indeed, these deficits have become characteristic of schizophrenia [50, 51]. These tasks all have unique associative bases [52, 53], and likely also involve attentional mechanisms [54–56]. However, what they have in common is that they all require the ability to filter out irrelevant information. For example, latent inhibition is the phenomenon whereby humans and other animals take longer to acquire a stimulus-reward association when the stimulus has previously been established as irrelevant by repeatedly presenting it alone (i.e., pre-exposure), which is thought to result in the development of a stimulus-no reward association [52, 57–60]. People with schizophrenia show faster rates of learning about pre-exposed stimuli and their associations with reward [61–68]. Importantly, clinical studies have shown that latent inhibition is also disrupted in otherwise healthy volunteers who score highly on measures of psychoticism and schizotypy [61, 63–66, 69], whilst medicated patients with schizophrenia show intact latent inhibition, indicating that this disruption is related to positive symptoms [62, 67, 70]. Moreover, the administration of haloperidol, a dopamine antagonist that treats psychotic symptoms in schizophrenia, has been shown to enhance latent inhibition in healthy participants [68].

Similarly, people with schizophrenia fail to show the blocking effect, a fundamental associative paradigm that involves both associative and attentional components [53–55, 71, 72]. Blocking involves first teaching subjects that a cue leads to reinforcement. Then, this predictive cue is paired with another, novel cue and the same reward. Here, blocking is evident when subjects do not learn about the novel cue, as it does not predict anything over and above the predictive cue and is deemed irrelevant [53, 54]. However, people experiencing acute, but not chronic, schizophrenia display deficits in blocking, characterised by enhancements in learning about the novel cue, during visual discrimination and spatial navigation tasks [73–80]. It was initially unclear whether the blocking deficit seen in patients was related to the negative or positive symptoms of the disorder. This was likely because task-related differences can change the dominant learning mechanism at play during blocking, resulting in either more reliance on a reinforcement learning [53], or on an attentional process [54]. Indeed, when the blocking task is accompanied by a general deficit in reinforcement learning in subjects with schizophrenia, poor blocking is associated with negative symptoms of the disorder [79, 80]. However, when the deficits in reinforcement learning are not present (due to changes in task structure or amounts of training), blocking is still impaired in people with schizophrenia, and is correlated predominantly associated with positive symptoms of the disorder and an inability modulate attention to the novel, blocked cue [74, 81]. This differentiates the blocking deficit in people with schizophrenia from that seen in latent inhibition, where a lack of latent inhibition in patients is attributed to disruptions in an associative process [52]. This makes sense as blocking and latent inhibition paradigms are dependent on different neural circuits (discussed below). These data demonstrate that the enhancement of learning about irrelevant cues in schizophrenia (including blocking and latent inhibition) correlates with positive symptoms of the disorder [81, 82], where it is thought that delusions and hallucinations that characterise positive symptoms arise as patients try to make sense of these aberrant learning experiences [83, 84].

On the other hand, people with schizophrenia display a consistent reduction in learning about cues that are predictive of reinforcement, which is thought to be related to the negative/cognitive symptoms of schizophrenia. Specifically, studies have reported relatively intact learning in people with schizophrenia when one cue-outcome contingency is available [85–96]. However, deficits are evident when complexities are introduced. For example, schizophrenia patients display robust deficits in reinforcement learning during probabilistic selection tasks [14, 29, 97–100], designed to assess a participant’s ability to learn from positive and negative feedback with changing probabilities of reinforcement [29, 101]. Even when patients are given an excess number of trials to learn probabilistic reinforcement contingencies, they still exhibit learning deficits, suggesting that deficiencies are the result of impaired learning from more complex rewarding outcomes, and not simply the result of slower stimulus-response learning, or basic working memory deficits that are frequently found in schizophrenia [97, 98]. With regards to reward-paired cues, these findings reveal that people with schizophrenia fail to make distinctions between events that are motivationally significant (e.g., rewarding), and display decreased updating of stimulus-outcome associations in response to changing reinforcement contingencies [83, 102, 103].

Collectively, these findings demonstrate a paradoxical deficit in schizophrenia; increases in learning about irrelevant stimuli, and a concomitant decrease in learning about reward-predictive information. Typically, these deficits are explained by changes in distinct neural circuits. For example, increases in learning about irrelevant stimuli are argued to result from increases in subcortical dopamine activity, supported by findings of elevated dopamine synthesis capacity in striatal regions specific to D2/3 receptors [104], which correlates with positive symptoms [31, 105]. On the other hand, reductions in learning about reward-predictive stimuli are often attributed to hypo-frontality, contributed to by a reduction in D1 receptor density, linked to negative symptoms and cognitive deficits [102, 106–108]. Studies using functional magnetic resonance imaging (fMRI) during reinforcement learning have corroborated this physiological evidence in some respects. For example, there is some evidence for hypo-frontality during learning in patients with schizophrenia [11, 82, 99, 109–111], though this does not appear to be the case for all frontal regions; while activity in some frontal regions is decreased, other show an increase in activity [112]. Further, fMRI data reveals that ventral striatal activity during reinforcement-learning tasks does not suggest an increase in dopamine function per se [82, 113]. Striatal activity is increased to irrelevant or neutral information and decreased to reward-predictive information [82]. Whilst this makes sense from a functional perspective of the schizophrenia learning paradox, it is difficult to reconcile a subcortical notion of hyperdopaminergic signalling, which would predict increased learning to both neutral and reward-paired cues. In the following sections, we will make the case that both positive and negative symptoms, and their corresponding deficits in reinforcement learning, instead fit within a cohesive model of dysfunction within specific dopaminergic circuits.

## Dopamine: a complex system subserving many different forms of reinforcement learning

**Fig. 1:**
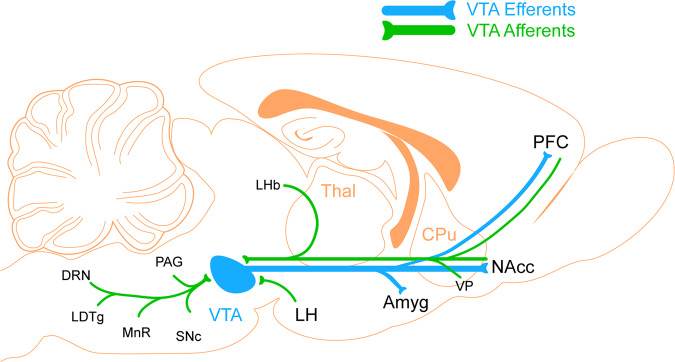
Schematic summarising the major inputs and outputs connecting with the ventral tegmental area (VTA) dopamine neurons. In the adult brain, dopaminergic neurons exist as a heterogenous group of cells localised predominately in the VTA and substantia nigra [190, 204, 205]. From these areas, dopaminergic projections arising from the VTA extend to limbic (mesolimbic) and cortical (mesocortical) regions, and from the substantia nigra to striatal (nigrostriatal), regions of the brain [206]. Given the overlap in common projections between the dopaminergic mesolimbic and mesocortical pathways, these two systems are often referred to the mesocorticolimbic pathway collectively [204] (Fig. 1). Mesolimbic and mesocortical pathways originating in the VTA send dopamine projections to the NAc and olfactory tubercle, and to limbic regions including the amygdala, hippocampus and frontal cortices through the medial forebrain bundle [207–209]. The VTA also sends and receives extensive reciprocal innervations from these same brain areas, as well as many others areas including the lateral hypothalamus, lateral habenula, dorsal raphe nucleus and periaqueductal grey [207–209]. In essence, dopamine neurons in the midbrain are densely connected with the rest of brain, where dopamine signalling is heavily influenced by descending projections, and in turn heavily influences processing in these regions to drive associative learning [34, 35, 39, 40, 44, 47, 118, 146, 210, 211]. Abbreviations: Amyg Amygdala, DRN Dorsal Raphe Nucleus, LDTg laterodorsal tegmentum nucleus, LH Lateral Hypothalamus, LHb Lateral Habenula, NAc Nucleus Accumbens, mPFC Medial Prefrontal Cortex, PAG Periaqueductal Grey, RMTg Rostromedial Mesopontine Tegmental Nucleus, VP Ventral Pallidum, VTA Ventral Tegmental Area.

To drive learning, dopamine neurons in the ventral tegmental area (VTA) exhibit a phasic error signal when an unexpected event has occurred [46, 114–116]. That is, dopamine neurons exhibit a signal that reflects the difference between what you thought was going to happen, and what happened in reality [46, 117]. This effectively instructs the brain to learn and update current expectations. Traditionally, this signal was only thought to contribute to what is referred to in the field as “model-free” learning [46]. This means that dopamine errors only instruct learning about something that has value, like food or money, allowing that numerical or scalar value to backpropagate to an antecedent cue [46]. However, recent studies have shown that this dopamine error acts more like a teaching signal to instruct humans and other animals to associate events together (e.g., stimulus-reward or stimulus-stimulus associations), regardless of whether either of those events contain something valuable or rewarding, and without endowing those events with value [33–35, 39–42, 118]. Further, dopamine errors in both humans and rodents contain information about predicted rewards [42], suggesting this signal serves to instruct neural regions on *what* to learn about, as well as *when* to learn [35]. This demonstrates that the dopamine prediction error does not act as a homogenous signal that broadcasts the value or salience of an event (or even allocations of lasting attention to a stimulus [43]), but as a teaching signal that is received throughout the brain to drive learnt associations that two constructs in the world are related (Fig. 1).

The evidence in favour of phasic dopamine acting as an instructive teaching signal to stamp in complex associations between events or actions are two-fold. The first comes from findings using optogenetic manipulation of VTA dopamine neurons during reinforcement learning [34, 39, 40]. Such studies have shown that stimulation of VTA dopamine neurons as a prediction error can facilitate the development of associations between predictive stimuli and their specific outcomes [34], and between two neutral sensory stimuli [39, 40], without endowing those antecedent stimuli with value [40]. Further, optogenetic inhibition designed to silence the dopamine prediction error across the transition between two neutral sensory stimuli, reduces the association between such stimuli [39], suggesting a physiological role for dopamine in the development of these associations. The second line of evidence supporting a role for dopamine in instructing learned associations comes from recording of neural activity in VTA (or nucleus accumbens) of mice, rats, and humans [42, 119, 120]. For instance, patterns of firing across ensembles of midbrain dopamine neurons have been shown to contain identity information of sensory prediction errors [42]. In addition, exciting new research has reported the presence of wave-like spatiotemporal dopamine dynamics in the dorsal striatum, with the propagation of specific wave trajectories (i.e., whether waves propagated from medial to lateral dorsal striatum, or lateral to medial) dependant on the demands of a learning task [119]. Both these findings suggest that spatiotemporal differences in dopamine signals may determine the timing and strength of subcircuit teaching signals, which ultimately defines what and when to learn. Consistent with this, regional differences in phasic dopamine responses within the striatum have been observed in response to novel cues [121], and predicted and unpredicted rewards [122, 123], with often opposing dopamine dynamics dependant on temporal aspects of the task and subjective experience. Together, this research demonstrates that the dopamine prediction error is not only capable of facilitating the development of specific sensory associations, but also that the information needed to learn these associations is present in the dopamine error itself, before it is received by the downstream circuit.

In this light, the paradoxical learning dichotomy seen in schizophrenia cannot be explained by a general increase in subcortical dopamine, nor an aberrant salience account, even with dissociable changes in the prefrontal cortex. This is because both these models would predict that deficits or enhancements in learning should occur in the same direction. For example, if people with schizophrenia exhibit a general increase in subcortical dopamine, then one would expect that learning about irrelevant or neutral cues, and reward-paired cues, would both be enhanced (or deficient) when this signal is altered, which is not observed in the clinical population. And in the aberrant salience model, it is hypothesised that chaotic dopamine firing leads to attribution of significance to stimuli that would otherwise be considered irrelevant [124–126]. However, the same chaotic firing should also enhance other aspects of learning at random, including learning from rewards. Maia and Frank attempted to resolve this by developing a model that described the subcortical dopaminergic changes occurring in schizophrenia as similar to what occurs in amphetamine [27]. Specifically, that an increase in dopamine could be exhibited spontaneously, enhancing sporadic associations that are linked to positive symptoms, but blunted to relevant information, which reduces learning about reward-paired cues. However, in light of the new data implicating the dopamine prediction error in learning about both neutral and reward-paired information, we would take this one step further. Specifically, we would argue that distinct dopamine circuits could encode learning about neutral information, and others could encode reward-related information, which is supported by emerging data [127, 128], and that the balance of these circuits could be changed in schizophrenia. Essentially, we believe it is likely that different circuits and brain regions utilise the dopamine prediction error as a qualitative teaching signal in specific ways, and that circuit-specific dysfunction in schizophrenia alters how the dopamine signal is received and interpreted, and ultimately the content and direction of what is learned. This would also be consistent with theories that propose more nuanced circuit-specific changes in dopamine function in schizophrenia could underpin the disparate symptomology observed in the disorder [31].

We would advocate for taking an approach that makes predictions from the reinforcement-learning deficits seen in schizophrenia as to the nature of the circuit-specific changes in dopamine circuits. The reasons for this are two-fold: (1) implicating circuit-specific changes in patients is hard to do in clinical research, owed to the lack of invasive techniques for probing biological characteristics in humans, and (2) we could then make predictions as to how manipulation of particular circuits in rodent models of schizophrenia could restore normal learning processing, with an ultimate goal of developing more targeted therapeutic compounds for the treatment of the disorder in humans. With that view in mind, we now know that dopamine signalling is both necessary and sufficient to drive associative learning between contiguous events, whether valuable or not [34, 38, 40, 43, 44, 118]. So, a model of the learning paradox in schizophrenia cannot assume a role for subcortical dopamine in learning about reward-paired information and not neutral information. Within this, we also know that different areas of the brain that interact with the dopaminergic circuits regulate learning about irrelevant, neutral, and rewarding information, respectively. For example, the prelimbic cortex of the rat, considered to be analogous to the dorsolateral prefrontal cortex (DLPFC) [129–133], regulates the allocation of attention to cues, facilitating performance in learned irrelevance tasks, overshadowing, and blocking [55, 56, 72]. Here, a reduction in prelimbic function in rodents- via lesion, functional inactivation, or dopamine depletion- produces deficits in attentional set shifting [134–137], blocking [55, 132, 138, 139], and overshadowing [55, 56], similar to what is reported in schizophrenia [81, 82, 87, 89–91, 140–142]. Indeed, people with schizophrenia show a reduction in DLPFC function during attentional set shifting and learned irrelevance [142–144], particularly those experiencing first-episode psychosis [145].

On the other hand, the orbitofrontal cortex is important for learning about the general structure of the environment, value-based decision-making, and goal-directed behaviour [33, 146–158]. This includes sensory-sensory associations, which extends to learning about associations between neutral stimuli [127]. The OFC is a functionally heterogenous structure divided into three prominent divisions: the medial orbital (MO), ventral orbital (VO), and lateral orbital (LO) cortices (with the LO overlapping with portions of insular cortex that share similar projection profiles, consistent with the human orbitofrontal cortex; e.g., [156, 159]). All three of these regions exhibit differential projection profiles and are thought to underlie distinct reinforcement-learning processes [159, 160]. In terms of relevance for our model, the encoding of sensory-sensory associations has been attributed to the LO. For instance, we recently found that optogenetic inactivation of LO reduced learning to associate neutral cues pairs [127], in a task very similar to that recently found to be disrupted in people experiencing hallucinations, including those with psychosis [28]. Further, reducing LO activity through lesions or inactivation in rodents also leads to an enhancement of latent inhibition [161, 162]. Such research demonstrates that OFC contributes in important ways to learning about neutral information, and that increases in OFC activity could produce an enhancement in learning about neutral information, and deficits in latent inhibition. Importantly, this has been supported by some imaging studies suggesting larger OFC volumes in people with schizophrenia [163, 164], and increases in OFC activity during reinforcement learning [112]. Finally, delusional ideation in healthy individuals has been associated with enhanced connectivity between the lateral OFC and visual cortex [165], where enhancements in updating beliefs about ambiguous neutral stimuli is correlated with the severity of positive symptoms in people with schizophrenia [166]. These findings demonstrate that overactivity in OFC circuits can enhance spurious associations about neutral stimuli, resulting in a bias towards prior experiences more heavily influencing future learning episodes, which may contribute to hallucinations and delusions. However, despite the extensive work looking at DLPFC and schizophrenia, there are fewer studies looking at OFC activity (and even less that dissect differential OFC subregions) in schizophrenia, particularly in the context of reinforcement learning, which make it difficult to draw concrete conclusions about the nature of OFC changes in schizophrenia.

In terms of learning about rewards, recent data has implicated the lateral hypothalamus as a novel structure that is critical to reward learning, and opposing learning about neutral or irrelevant information. The lateral hypothalamus has long been implicated in responding to rewards [167–176], and recently this has been extended to biasing learning towards the predictors of rewards [128, 175]. For instance, optogenetic inhibition of GABAergic neurons in the lateral hypothalamus decreases learning of reward-predictive cues, whilst enhancing associations formed between neutral cues and abolishing latent inhibition [128, 175]. Importantly, the lateral hypothalamus is a very diverse region and contains many other neuronal populations that have been implicated in motivated behaviour [177–184]. Thus, it is likely that many neuronal populations within the lateral hypothalamus contribute to these effects on learning. It may be that the GABAergic neurons receive information from the many distinct populations within the lateral hypothalamus, which is then relayed to other neural structures via the dense projections that GABAergic neurons in this region send throughout the brain, to influence ongoing learning and behaviour [175]. These data implicate the lateral hypothalamus, and GABAergic neurons in particular, as a critical arbitrator of learning about reward-predictive information and neutral information, and are strikingly similar to the paradoxical deficits we see in schizophrenia [27, 29, 30, 81, 113]. That is, changes in hypothalamic activity in people with schizophrenia has the capacity to change the balance in learning about reward-paired and neutral stimuli, producing both sides of the reinforcement learning deficits of the disorder. However, there are very few imaging studies looking at hypothalamic changes in schizophrenia, and those that have looked at hypothalamus in people with schizophrenia have elicited mixed results [185–187]. This is likely related to the difficulty in imaging this structure, requiring manual quantification [188, 189]. Given there are few studies investigating the potential for hypothalamic dysfunction in schizophrenia, and no studies that have looked at lateral hypothalamic dysfunction in the context of the reinforcement-learning deficits seen in schizophrenia, this is a particularly promising direction for future research.

So then, how might we reconcile these region-specific roles in the reinforcement learning deficit seen in schizophrenia to put forward a new theoretical framework? The circuits comprising dopamine neurons and the prelimbic cortex, orbitofrontal cortex, and lateral hypothalamus are complex [72, 131, 175, 176, 190]. However, from a functional perspective, a particularly interesting locus for parallel and/or competitive interactions between these systems could be the nucleus accumbens in ventral striatum. The nucleus accumbens receives dense projections from midbrain dopamine neurons, and is generally considered a proxy for midbrain dopamine activity [170, 191–196]. Indeed, most of the imaging data in reinforcement-learning studies in the schizophrenia literature look at activity in ventral striatum [82, 83, 113] (but see [31] for evidence in changes in the dorsal striatum).

Importantly, prelimbic cortex, orbitofrontal cortex, and lateral hypothalamus all send projections to different areas of the accumbens [175, 197, 198], and the areas of the nucleus accumbens that receive these projections appear to be functionally distinct [162, 199–201]. For example, the prelimbic cortex sends dense projections to the medial portions of the nucleus accumbens core (and to a lesser extent, shell) [198]. This same portion of the nucleus accumbens has been implicated in the attentional mechanisms of the blocking and overshadowing effects, which are also dependent on the prelimbic cortex [55, 56, 132, 138, 139, 202, 203]. The orbitofrontal cortex projects to the nucleus accumbens core, though a more lateral region from that receiving projections from prelimbic cortex [198]. A reduction in activity in this portion of the ventral striatum produces enhanced latent inhibition [162, 200], similarly to a loss of function in OFC [161, 162]. Further, neurons in nucleus accumbens core also reflect learning of neutral associations [187], where this form of learning is known to be dependent on OFC function also [127]. Finally, GABAergic neurons in the lateral hypothalamus project to yet another region of the ventral striatum, the shell of the nucleus accumbens, critical for responding to specific cue-reward associations and latent inhibition [200]. Thus, prelimbic, orbitofrontal, and lateral hypothalamic areas all project to distinct regions of the nucleus accumbens that appear to mirror the roles in learning subserved by these afferent structures.

**Fig. 2:**
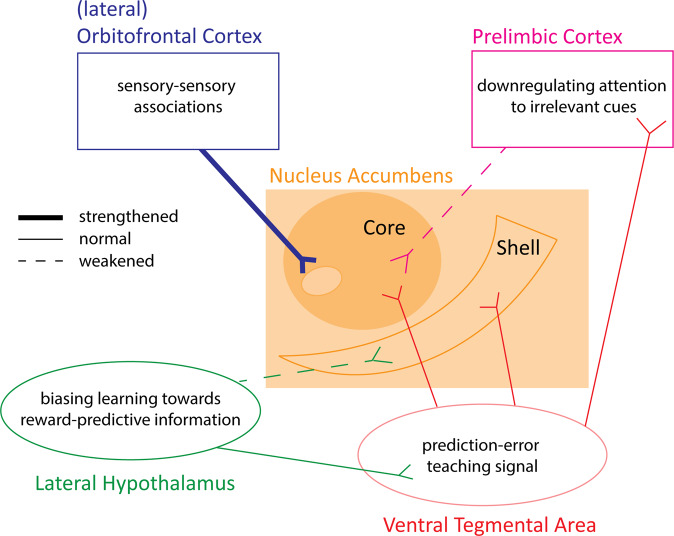
A hypothesised framework of how distinct dopaminergic circuits could be altered in schizophrenia. People with schizophrenia show a learning paradox, characterised by increases in learning about neutral or irrelevant information, and a decrease in learning about reward-predictive information. This is associated with an increase in activity in ventral striatum to neutral or irrelevant information, and a decrease in activity to reward-predictive information [27, 29, 30, 81, 113]. One way to account for this deficit is to hypothesise that the inputs to the ventral striatum that regulate the balance between learning about reward-predictive, neural, and irrelevant information, are changed. Specifically, we would argue that the paradox seen in schizophrenia is consistent with a strengthening of orbitofrontal inputs to the ventral striatum, and a decrease in the inputs from prelimbic and lateral hypothalamic circuits. The feasibility of such a model is supported by the separable nature of afferents coming from these regions to ventral striatum [156, 159, 175, 197, 198]. Further research is necessary to test the validity of this model using preclinical studies in the context of reinforcement learning.

To put forward a new framework then, it may be the case that the prediction error from VTA dopamine neurons terminating in the nucleus accumbens serves to facilitate learned associations in the form of synaptic plasticity [205, 212, 213], and these sites receives a form of top-down modulation by afferents from prelimbic, orbitofrontal, and lateral hypothalamic inputs. These top-down afferents could compete to influence the balance of learning between predictive, neutral, and irrelevant information. In reference to schizophrenia, it may be that these circuits are differentially weighted, such that the circuits comprising orbitofrontal cortex and ventral striatum are overweighted relative to prelimbic and hypothalamic striatal circuits, which would produce an increase in learning about neutral and irrelevant information, while reducing learning about reward-predictive cues. This could occur in at least two ways: (1) the inputs from the orbitofrontal, prelimbic, or lateral hypothalamic region onto ventral striatum could be changed, or (2) the bottom-up inputs from ventral tegmental area onto different portions of ventral striatum could be differentially changed (Fig. 2). Overall, this view would be in line with clinical evidence suggesting that dopamine receptor blockers, whilst somewhat effective against positive symptoms, offer little to ameliorate (and can even exacerbate) negative symptoms [214]. Specifically, by building on recent models [27], this could be because dopamine receptor antagonists exacerbate the already attenuated phasic dopamine responses in the ventral striatum that underpin reward learning and are facilitated by prelimbic cortex and hypothalamic inputs. On the other hand, this would be effective in reducing phasic dopamine responses in overactive circuits comprising OFC that contribute to the increase in learning about neutral information, which are theorised to underlie positive symptoms. In the below section, we discuss the research that is needed to investigate these possibilities. If our hypothesis is supported by future work, this would mean that pharmacotherapies targeting dopamine need to be designed to modulate dopamine differentially in distinct circuits in order to combat both positive and negative symptoms of the disorder (but see promising effects of deep brain stimulation in dopamine circuits, which may influence dopamine circuits through multiple mechanisms; [215]).

## A “call to action”: the need for studies using preclinical models of schizophrenia in the context of reinforcement learning

**Fig. 3:**
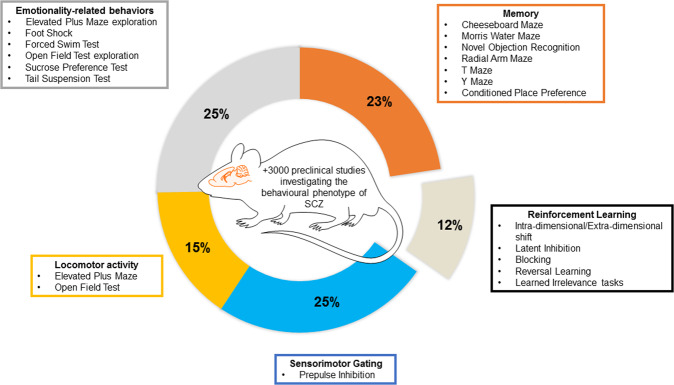
Graphical representation of behavioural domains commonly investigated in preclinical models of Schizophrenia. The sensorimotor assay described as the prepulse inhibition test makes up the single most cited test in preclinical schizophrenia research. Other behavioural assays include locomotor activity, and emotionality-related behaviour in the open field test, elevated plus maze, forced swim test, sucrose preference test, tail suspension test and foot shock aversion test. Working, spatial and reference memory contribute to the majority of behavioural assays designed to test memory that are cited by preclinical research, with the Morris water maze, cheeseboard maze, radial arm maze, T maze, Y maze and novel object recognition the most commonly cited. Reinforcement-learning tasks, which make the most contact with the clinical literature, comprise only 12% of citations, which includes latent inhibition, reversal learning, reinforcement learning and intradimensional/extradimensional set-shift tasks that have been directly tested in people with schizophrenia.

Preclinical models of schizophrenia are particularly useful for studying the neural underpinnings of particular reinforcement-learning tasks and how a change in particular circuits could produce learning deficits that mirror what we see in this disorder. Indeed, in the 80 s and 90 s there was a concerted effort to use reinforcement-learning tasks like latent inhibition and blocking in tandem with drugs that target dopamine systems to mimic the deficits seen in schizophrenia [51, 57–59, 216, 217]. This literature served as a cornerstone for understanding the neural bases of the early deficits seen in reinforcement learning in schizophrenia, and were some of the first to provide support for the dopamine hypothesis of schizophrenia [58, 217]. However, in the last few decades there has been a shift in the field away from using preclinical models in the context of reinforcement learning, towards assays that assess emotionality-related behaviours, such as those concerned with anxiety-like phenotypes and learned helplessness or behavioural despair, and hyperactivity and sensitivity to psychotomimetic drugs [218–220]. Those studies that do assess learning-related phenomena typically implement behavioural assays specific to working, reference and spatial memory (e.g., Cheeseboard, Morris Water, Radial Arm, T- and Y-mazes), many of which do not map onto tasks used in clinical populations. In Fig. 3, we represent a breakdown of paradigms used in the field, sampling for over 3000 preclinical studies using rodent models of schizophrenia. This summary gives a general representation of the state of the current field^1^. This clearly shows that reinforcement-learning tasks, which can be directly replicated in humans and are closely related to both the positive and negative/cognitive symptoms of schizophrenia, fall behind in relation to tasks that assess basic emotional reactivity.

Another challenge facing the literature is that it is difficult to find a preclinical model of schizophrenia that exhibits changes consistent with both the positive and negative/cognitive symptoms of schizophrenia. For example, most preclinical models of schizophrenia in the early years were based on dopamine agonists, like amphetamine, or glutamate (NMDA) antagonists, like PCP and MK-801 [221–224]. Those that enhance dopamine signalling found increases in attention or learning about irrelevant information [217, 225–231]. On the other hand, those reducing glutamate signalling tended to find changes consistent with negative/cognitive symptoms, including reversal learning [232–235], associative fear learning [236] and working memory [237–239]. However, the advent of genetic neuroscience has the potential to provide models that more closely mimic the human disorder, which may prove to show deficits consistent with both sides of the learning paradox seen in people with schizophrenia. For example, there is now a rodent model of the 22q11.2 deletion syndrome, considered to be the strongest copy number variant occurring in the human population that is directly linked to schizophrenia, accounting for 1 in 100 cases [240]. People with the 22q11.2 deletion show significant deficits in learning consistent with schizophrenia, including those consistent with changes in predictive coding, working memory, reward processing, and pre-pulse inhibition, some of which has now been replicated in the 22q11.2 rodent model [241, 242]. While early days, models like the 2211.2 deletion could be used in combination with emerging tools that can exert specific control over neuronal populations to test some of the prediction outlined in our framework (Fig. 2), discussed below.

## New tools and approaches for investigating Schizophrenia

**Fig. 4:**
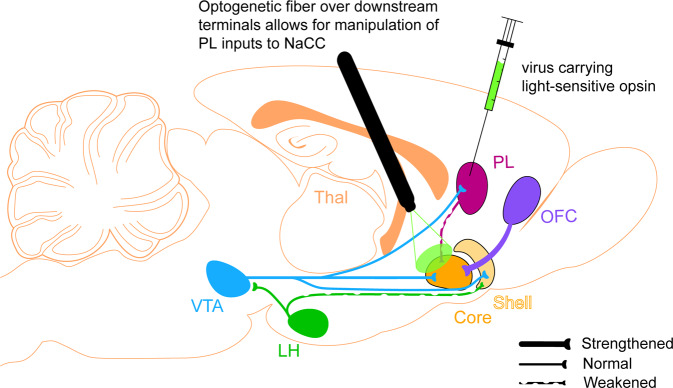
New genetic tools facilitate circuit-specific manipulation and recording of dopamine activity and related circuits in vivo. The advent of optical methods for manipulating and visualising cell-type specific neuronal activity have revolutionised behavioural neuroscience and provide critical means with which to record and manipulate activity in specific dopamine circuits. This paves the way to investigate (1) how different dopamine circuits contribute in unique ways to associative learning, and (2) how specific dopamine circuits in rodent models of schizophrenia may be altered, contributing to the learning paradox seen in the disorder. For instance, one way to test the hypothesis that the inputs to NaCC from the prelimbic cortex are weakened in schizophrenia, would be to inhibit prelimbic terminals in NAcc and determine the resultant effect on attentional related process. One could then compare the phenotype produced by such manipulation to that of schizophrenia to elucidate neural substrates underpinning learning deficits. Abbreviations: LH Lateral Hypothalamus, NAcc Nucleus Accumbens, OFC Orbital Frontal Cortex, PL Prelimbic Cortex, VTA Ventral Tegmental Area.

Pharmacological studies have been invaluable in schizophrenia research. However, they are limited by the fact that pharmacological agents alter dopamine signalling over extended timescales, and as a result, cannot directly be linked to specific patterns of neuron activity [118, 243, 244]. However, the optogenetic revolution now affords manipulation of particular neuronal populations and their projections to distinct regions of the brain with millisecond temporal resolution [243, 244]. The importance of this development cannot be overstated. For example, the use of transgenic rodents expressing Cre recombinase under the control of the tyrosine hydroxylase (TH-Cre^+^) or glutamate decarboxylase (GAD-Cre^+^) promoter, allows for the Cre-dependent expression of opsins such as channelrhodopsin-2 (ChR2) or halorhodopsin (NpHR) in dopamine or GABA neurons, respectively [175, 211, 245]. The expression of ChR2 and NpHR in these rats would allow for the ability to activate and inhibit, respectively, dopamine or GABA neurons with millisecond resolution in behaving rodents [118, 243, 244, 246, 247]. Importantly, the Cre-dependent expression of these opsins in the cell body will also travel in the anterograde direction along the axons of the cell body, reaching the afferent terminals of the region receiving those afferents [243]. This allows inhibition of downstream terminals while leaving upstream neuronal cell bodies intact (Fig. 4) [245]. Cell-type specific optogenetics is a particularly useful tool in the context of schizophrenia, as it allows for targeting of particular dopaminergic circuits that underlie different aspects of reinforcement learning, which could elucidate the ways in which these circuits are likely to be affected in the disorder. Whilst there are technical challenges yet to be overcome before optogenetics are applied for therapeutic purposes in the clinic [248], advances for tracing neural connections in humans (i.e., diffusion tensor imaging; [249]), provides a parallel in which disrupted circuits identified in the clinic can be probed using optogenetics in animal models. For instance, endophenotypes commonly observed in schizophrenia patients could be precisely replicated in rodents, and tested to determine if aberrant changes in neural circuits play a causal role in specific symptoms [250, 251]. These changes could then be used as biomarkers in preclinical drug screening to determine the efficacy of novel therapeutics, which could rely on genetic approaches more amenable to clinical trials, like Designer Receptors Exclusively Activated by Designer Drugs (DREADDs) [250–252].

Similar optical approaches can be used to image activity in particular neuronal populations, or the image release of particular neuro-modulatory chemicals in vivo at high spatiotemporal resolution [245]. For example, fibre photometry, or single-photon imaging using miniscopes, can be used to image calcium activity in dopamine neurons. Specifically, injection of either a Cre-dependent AAV carrying the calcium sensory GCaMP into a TH-Cre rat can facilitate imaging of activity in dopamine neurons [245, 253]. Further, the very recent development of several genetically encoded dopamine sensors [e.g., dLight, G-protein-coupled receptor-activation-based (GRAB) DA] enables rapid optical recording of dopamine dynamics without the use of transgenic rodents [254–256]. This may be particularly advantageous for investigating changes in dopamine signalling seen in genetic models of schizophrenia that are not usually founded on a Cre line and therefore not as easily used in combination with Cre-dependent optogenetic manipulation and calcium imaging of dopamine neurons. While dopamine sensors do not facilitate imaging of projection-specific dopamine release itself, it can also be used to detect physiological relevant dopamine transients caused by optogenetic manipulation of particular terminals, or adjacent to terminals that have been tagged by viral vectors [254]. These techniques constitute a veritable arsenal for investigating how activity in dopaminergic circuits influences reinforcement learning, and how these dynamics may be changed in preclinical models of schizophrenia.

## Conclusions and remaining questions

Recent evidence has changed the way we think about the dopamine prediction error from a functional perspective [33, 34, 39–43, 118, 257]. We now know that the dopamine error acts as a teaching signal to instruct the development of learned associations in many different contexts not traditionally envisioned by theories of dopamine function, including learning about neutral information. Accordingly, we may now be able to garner a better understanding of how dysfunction in specific dopamine circuits could contribute to the reinforcement learning paradox seen in people with schizophrenia. Here, we argue that this paradox can be accounted for by an imbalance in the circuits that utilise the dopamine prediction error signal in the nucleus accumbens to stamp in distinct learned associations. We hypothesise that circuits comprising orbitofrontal modulation of nucleus accumbens are strengthened, heightening the development of associations between neutral information. On the other hand, we argue that prelimbic and hypothalamic inputs to the nucleus accumbens are compromised relative to orbitofrontal inputs, reducing the ability of people with schizophrenia to modulate attention and learning towards stimuli in the environment that are the best predictors of outcomes. There are many questions that remain for this theoretical model. For example, in the current manuscript, we have focussed on the potential contribution of phasic dopamine signals to the deficits in schizophrenia. However, levels of tonic dopamine play integral roles in general motivation and decision making, and are in some cases distinct (or modulatory) to the phasic error signal [258–260]. Thus, future discussions of how particular dopamine circuits contribute to the reinforcement-learning deficits seen in schizophrenia should comprise discussion of potential changes in tonic dopamine function. In addition, there is evidence for dysfunction in other regions of the brain seen in schizophrenia than those discussed here. For example, studies have shown that function is compromised in the ventromedial prefrontal cortex of people with schizophrenia (vmPFC; considered analogous to infralimbic cortex in the rat [131, 132, 261]). This region also sends dense projections to the nucleus accumbens shell [198], and plays important roles in reinforcement learning, which may suggest an integral role in the deficits seen in schizophrenia and our framework presented here. Recent technological advances have granted new avenues for investigating cell-type specific activity in cell bodies and their projections. By using these techniques, we can now explore how projection-specific activity contributes to learning deficits seen in animal models of schizophrenia. Importantly, these techniques will help identify circuit-specific biomarkers of the disorder not previously envisioned by current models, which could be used in preclinical drug screening. Ultimately, this approach could pave the way for more novel therapeutics targeting dopamine activity that could prove efficacious for both positive and negative/cognitive symptoms.

## Footnotes

1 (A Web of Science indexed search was performed using the following keywords: “Schizophrenia AND/OR Open Field Test, Elevated Plus Maze, Forced Swim Test, Morris Water Maze, Cheeseboard Maze, Radial Arm Maze, T Maze, Y Maze, PPI, Sucrose Preference Test, Tail Suspension Test, Novel Object Recognition, Foot Shock, ID/ED, Latent Inhibition, Reversal Learning, Conditioned Place Preference, Reinforcement Learning”. Keywords “Rat OR Mice” were used to exclude clinical studies. Only original manuscripts were considered, reviews were excluded from the search).
